# scRiskDB: a single-cell epigenomic resource linking complex traits to regulatory mechanisms across human tissues

**DOI:** 10.1093/database/baag048

**Published:** 2026-08-05

**Authors:** Gefei Zhao, Xianfeng Ping, Binbin Lai

**Affiliations:** Institute of Medical Technology, Peking University Health Science Center, Beijing 100191, China; Biomedical Engineering Department, Institute of Advanced Clinical Medicine, Peking University, Beijing 100191, China; Central Laboratory, Peking University School and Hospital of Stomatology & National Center for Stomatology & National Clinical Research Center for Oral Diseases & National Engineering Research Center of Oral Biomaterials and Digital Medical Devices, Beijing 100081, China; Institute of Medical Technology, Peking University Health Science Center, Beijing 100191, China; Biomedical Engineering Department, Institute of Advanced Clinical Medicine, Peking University, Beijing 100191, China; Department of Dermatology, Peking University First Hospital, Beijing 100191, China; Peking University International Cancer Institute, Beijing 100191, China

## Abstract

Understanding the regulatory impact of non-coding genetic variants remains a major challenge in human genetics. Here, we present scRiskDB, a comprehensive and user-friendly database that maps genetic risk variants to their downstream regulatory elements, target genes, and relevant cell types at single-cell resolution. By integrating genome-wide association studies (GWAS) with single-cell datasets across 45 tissues and developmental stages, scRiskDB implements a variant-to-function framework that systematically outlines potential regulatory cascades from single nucleotide variants to cell-specific risk mechanisms. This multi-layered design allows users to explore trait-associated regulatory architectures across cell types and developmental stages. The platform provides interactive, multi-level visualizations and curated results, facilitating hypothesis generation and mechanistic insights into disease aetiology.

## Introduction

Understanding the genetic basis of complex traits and diseases requires resolving the regulatory mechanisms by which non-coding variants influence gene expression in a cell-type-specific manner. Although genome-wide association studies (GWAS) have identified thousands of trait-associated variants, the vast majority variants lie in non-coding regions, making it difficult to interpret their functional consequences [1–3]. Recent advances in single-cell technologies offer an unprecedented opportunity to link these variants to their regulatory targets with high resolution and cellular specificity [4, 5].

**Figure 1:**
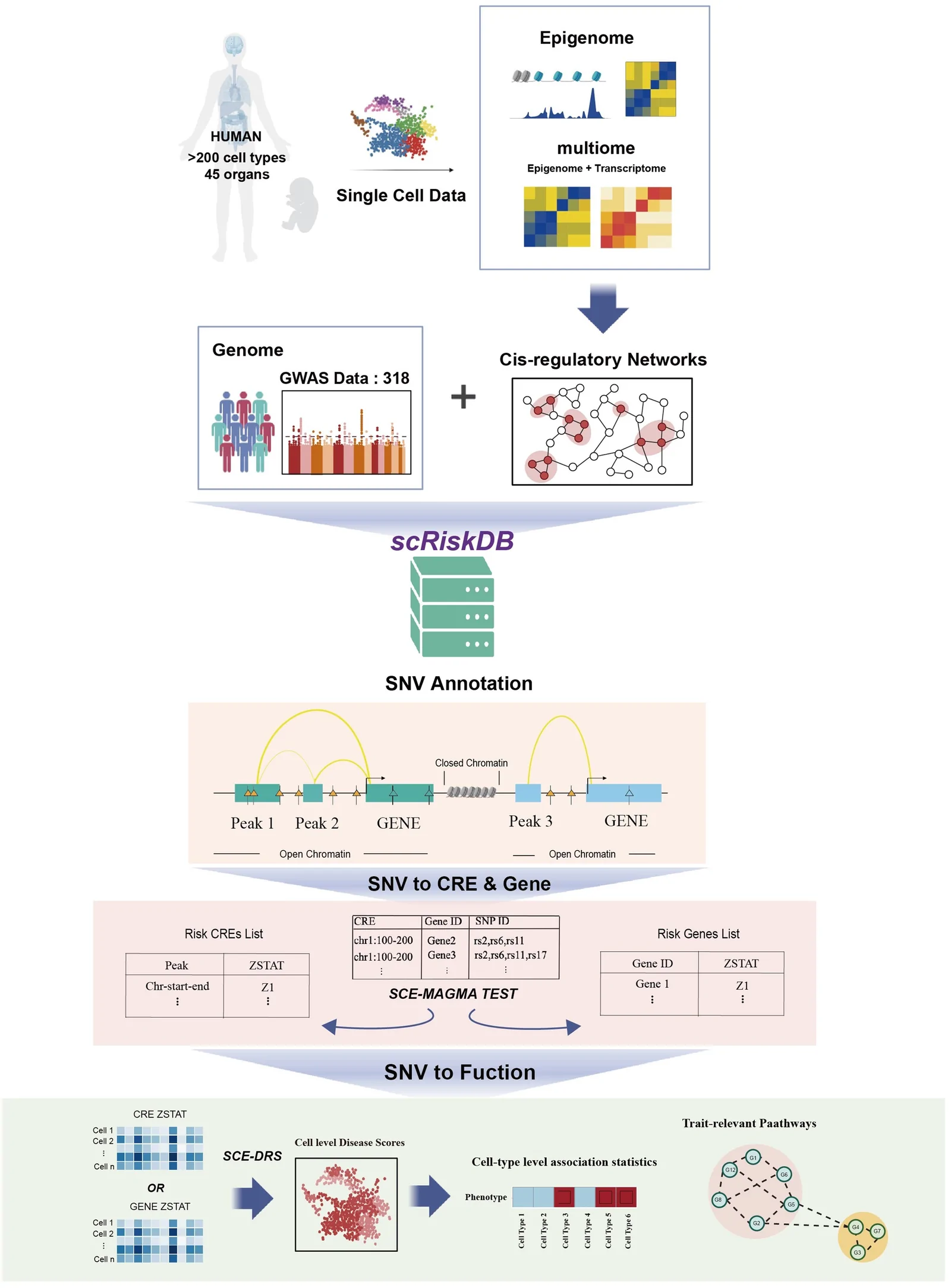
Variants to SNV framework of scRiskDB. scRiskDB implements a systematic variant-to-function framework that integrates single-cell data and GWAS data to functionally interpret disease-associated single nucleotide variants (SNVs). The platform compiles multimodal single-cell datasets across over 200 human cell types and 45 organs. Trait associations are quantified at the levels of *cis*-regulatory elements (CREs) and genes using the sce-MAGMA framework and projected onto the single-cell landscape via sce-DRS. The architecture of scRiskDB encapsulates its central logic: mapping SNVs to CREs and genes, and ultimately to their functional impacts in specific cellular contexts.

Here, we present scRiskDB, a web-accessible, registration-free database that connects GWAS signals to functional regulatory elements at single-cell resolution. By integrating regulatory networks, genome annotations, and epigenomic information from single-cell datasets, scRiskDB enables identification of disease-associated genes and *cis*-regulatory elements (CREs) across diverse tissues and developmental stages (Fig. 1). A key strength of scRiskDB lies in its variant-to-function framework, which systematically traces the full flow of information across multiple biological layers—from single nucleotide variants (SNVs), to CREs, to genes, and finally to specific cell types. Importantly, scRiskDB is designed as an integrative annotation and prioritization resource. The regulatory links provided represent putative associations inferred from epigenomic context and genetic association signals and are intended to support downstream prioritization and experimental follow-up. This integrative, multilayered approach allows for comprehensive dissection of regulatory cascades underlying complex traits and offers novel insights into disease mechanisms. To our knowledge, no existing resource provides such a comprehensive mapping of GWAS signals to regulatory elements and cell types using single-cell epigenomic data.

Compared to existing databases such as EnhancerAtlas [6], SEdb [7], and GeneHancer [8], which rely on bulk epigenomic data and thus reflect average signals across heterogeneous cell populations, scRiskDB captures cell-specific regulatory dynamics by leveraging single-cell data. While more recent tools like scEnhancer [9] use single-cell epigenomic data to annotate enhancers, they lack integration with GWAS findings. Conversely, tools like sc2GWAS [10] link GWAS loci to cell types using single-cell transcriptomics but do not incorporate CRE-level data or enable cross-stage comparisons. scRiskDB uniquely integrates both genetic association and single-cell epigenomic data, enabling cross-layer, cross-tissue, and cross-stage exploration of trait-relevant regulatory architecture.

The current release of scRiskDB includes data from 45 human tissues spanning both adult and foetal stages, over 200 annotated cell types, and integrates GWAS summary statistics for >300 complex traits and diseases. This extensive coverage enables in-depth investigation of the cell-type-specific regulatory basis of human diseases, supporting both hypothesis generation and exploratory data analysis. Built-in analytic tools and a streamlined interface further empower users to explore trait-relevant genes, CREs, and pathways, or conduct custom analyses using their own single-cell datasets.

By combining genetic association data with the regulatory architecture revealed by various single-cell data, scRiskDB serves as a powerful resource to elucidate the molecular underpinnings of complex traits and to identify candidate regulatory mechanisms that may inform disease risk and therapeutic targeting.

## Material and methods

### Implementation of scRiskDB

The scRiskDB platform is deployed on a CentOS 7.9.2009 and is accessible via www.scriskdb.cn. The system is built entirely using R (version 4.3) and is hosted via Shiny Server (https://posit.co/products/open-source/shiny-server/), a web server specifically designed to serve interactive Shiny applications. The web interface is developed using the shiny dashboard framework, which provides a structured and responsive user interface for visualizing and querying the database. The shiny-based framework code for web builds is available on GitHub (https://github.com/gefeiZ/scRiskDB).

All data storage and retrieval operations are managed by a MySQL relational database (version 8.0.29), which serves as the core data backend. To ensure secure remote access to the internal database, Fast Reverse Proxy is configured for encrypted tunnelling, enabling safe communication across network boundaries. The Shiny Server is reverse proxied through Nginx (version 1.20.1), which handles HTTP requests, static file delivery, and supports SSL termination to ensure secure web access. This architecture enables seamless integration of interactive analytics with robust server-side computation and data storage. Users are encouraged to access the platform through modern web browsers that support HTML5 standards, such as Google Chrome or Microsoft Edge, for an optimal experience.

### Single-cell data collection and integration

We collected atlas-level single-cell datasets with cell-type annotations across 45 tissues spanning both foetal and adult developmental stages. The Chromatin accessibility profile (scATAC-seq) was used as the primary modality for all tissues. Additionally, to capture more complex dynamics, we incorporated a foetal-to-adult brain atlas generated using 10x Single Cell Multiome technology (scRNA-seq and scATAC-seq). The single-cell ATAC datasets include profiles from 16 foetal and 28 adult organs, covering >200 annotated cell types. Detailed information for all public single-cell datasets used in this study can be found in Supplementary Table S1. All samples were aligned to the GRCh38 reference genome. Data processing was performed independently for each tissue to preserve tissue-specific biological signals.

We implemented a stringent quality control strategy for both modalities following standard recommendations from the Seurat [11] and Signac [12] analysis frameworks. We restricted downstream analyses to peaks mapped to standard chromosomes. To address peak definition inconsistencies across samples, we adopted a unified feature set strategy in line with established Signac best practices. For each tissue, we first generated a comprehensive set of genomic regions by aggregating peaks across all samples. Peaks with widths between 20 and 10 000 base pairs were retained. Cells with <1000 fragments were removed, and only cell types represented by at least 10 cells were retained. We then use the reduce function from GenomicRanges [13] to merge overlapping peaks into a consensus set of non-overlapping genomic regions. The raw fragment counts for all samples within a tissue were then re-quantified against this unified peak set, ensuring a standardized feature space for downstream analysis.

We performed dimensionality reduction using Latent Semantic Indexing (LSI). For each dataset, we set the peak signal to 1 (open) if at least one read was present and to 0 (closed) if no reads were present. The binary peak-cell matrix was converted into a Term Frequency-Inverse Document Frequency (TF-IDF) matrix, followed by Singular Value Decomposition (SVD) to extract latent features. For the gene expression modality available in the brain dataset, cells were filtered to retain those with detected RNA counts between 200 and 50 000 and a mitochondrial read percentage of <5%. Gene expression data were normalized and scaled using SCTransform to remove technical variation. And the Principal Component Analysis (PCA) method was performed for dimensionality reduction.

### Public GWAS data collection and processing

We collect publicly available GWAS summary statistics from the FinnGen [14] consortium (Release R11), one of the largest biobank-based GWAS resources to date. FinnGen was chosen for its large sample size, broad disease coverage, and systematic phenotype curation based on standardized diagnostic codes, making it particularly suitable for cross-tissue and cross-cell-type analyses of genetic risk.

We downloaded the complete set of GWAS summary statistics from the FinnGen R11 release. To ensure phenotypic consistency and clinical interpretability, we retained only those datasets corresponding to diseases explicitly mapped to standardized ICD-11 codes. GWAS entries lacking clear ICD-11 annotations were excluded. Following this filtering step, a total of 318 GWAS summary statistics were retained for downstream analyses (Supplementary Table S2). We uniformly GWAS summary statistics data into standard P-value formats comprising four columns of information (dbSNP ID, chromosome, position, and *P*-value). As publicly available GWAS summary statistics were used, no data points were excluded from analysis, and no statistical methods were employed to predetermine the sample size.

### Identification of *cis*-regulatory interactions in single-cell data

Multi-omics and epigenomic datasets offer a powerful means to resolve cell-type-specific regulatory networks, enabling the identification of putative *cis*-regulatory interactions with high resolution and context specificity. To systematically map such interactions, we leveraged LinkPeaks function in Signac package [12] and Cicero [15] tool.

For single-cell multiome datasets, we employed the LinkPeaks function implemented in the Signac R package to identify peak–gene associations. This function models the association between accessibility at distal peaks and the expression of nearby genes across individual cells using correlation-based methods. Only statistically significant associations (adjusted *P*-value < .05) were retained for downstream analysis.

For scATAC-seq datasets, we used the Cicero [15] method to infer *cis*-regulatory interactions based on co-accessibility patterns. Using the latent features derived from LSI as we described above and UMAP embeddings, we computed co-accessibility scores within defined genomic distances. The co-accessibility network was constructed using a graphical LASSO framework. Peak pairs with Cicero co-accessibility scores >0.1 were retained as putative regulatory interactions. This threshold was selected based on commonly adopted practices in previous studies using Cicero and was intended to balance sensitivity and specificity when inferring regulatory interactions from sparse single-cell chromatin accessibility data. Moderate co-accessibility thresholds help preserve candidate distal enhancer–promoter interactions, which often exhibit weaker correlation signals compared with promoter–proximal interactions. To further control for potential false-positive interactions, downstream analyses incorporated additional biological constraints, including tissue-specific chromatin accessibility and trait-relevant gene prioritization. Together, these approaches allowed us to construct a comprehensive candidate *cis*-regulatory interaction landscape across diverse tissues and cell types.

### Generation of epigenome-based trait-relevant SNV annotation

To annotate trait-associated SNVs, we employed an epigenome-informed strategy SC-VAR: sce-MAGMA [16], which integrates both genic and regulatory contexts. Each SNV was assigned to genes through two complementary mechanisms. First, SNVs located within the gene body—including exons, introns, and untranslated regions (UTRs)—were directly linked to the corresponding gene. Second, non-coding SNVs reside within CREs were indirectly assigned to genes based on regulatory interactions. These regulatory associations were inferred from the *cis*-regulatory networks described above. Both categories of SNVs were merged to construct a comprehensive SNP-to-gene annotation file. Redundant SNV–gene pairs were removed to avoid inflation in downstream analyses. The resulting annotation captures both coding and non-coding variants potentially influencing gene regulation and expression, thus providing a more complete map of functional variant–gene relationships.

### Identification of trait-relevant risk genes and *cis*-regulatory elements

To identify trait-relevant risk genes, we applied the sce-MAGMA gene analysis module, using the previously constructed SNP-to-gene annotation file. In brief, gene-level associations were calculated using a multiple linear principal component regression model based on the gene-SNP matrix, and statistical significance was assessed via *F*-tests to derive gene-level *P*-values. Then the *P*-values were further transformed into *Z*-scores, which we refer to as ZSTAT, reflecting the strength of each gene’s association with the trait or disease. For the identification of trait-relevant CREs, we employed the SC-VAR: sce-DRS [16] module. This method identifies risk regulatory elements that are associated with risk genes using a Peak-SNP-Gene annotation. And took the largest SNP *Z-*score under the peaks as the CRE ZSTAT. These analyses enable systematic prioritization of genes and CREs enriched for trait-associated genetic signals, thereby providing an epigenetically informed view of genetic risk architecture across tissues and cell types.

### Pathway enrichment analysis

To identify biological pathways enriched among disease-associated genes, we performed over-representation enrichment analysis using the clusterProfiler [17] R package. This method statistically evaluates whether genes from predefined functional categories are present more frequently than expected by chance in each subset of genes. Specifically, we applied it to the set of genes associated with trait-related CREs as identified through scRiskDB. For the functional category, we focused on Gene Ontology Biological Processes (GO: BP) by setting the ont = ‘BP’ parameter in the enrichGO function. Significance of enrichment was assessed using the hypergeometric test, and *P*-values were adjusted for multiple comparisons using the Benjamini–Hochberg method. To visualize the users selected enriched pathways, we used the dotplot function from clusterProfiler. Dot size corresponds to gene count, and colour indicates the significance level. This allowed for intuitive comparison of enriched biological processes potentially involved in the trait or disease of interest.

### Identification of trait-related cell association

To identify trait-related cells, we employed the sce-DRS module from the SC-VAR method. We constructed risk files in the format required by sce-DRS, using either the trait-relevant risk genes or risk CREs identified from previous analyses. These files served as the input to sce-DRS, which computes two key metrics at the single-cell level: (1) a disease association *P*-value, and (2) a normalized disease relevance score, quantifying the enrichment of disease-associated signals within each cell.

Cell-level associations were subsequently aggregated to assess associations at the level of cell clusters, cell types, or other biologically meaningful groupings. This enabled context-specific identification of trait-relevant cellular populations across multiple tissues and developmental stages. To further characterize the regulatory landscape of these disease-relevant cells, we applied EpiScanpy [18] to identify cell type-specific trait-associated CREs. To ensure the cell-type specificity of the regulatory mechanisms, we implemented a strict filtering strategy: we intersected the tissue-level *cis*-regulatory network with these cell-type-specific open peaks. A risk CRE and its target gene were assigned to a specific cell type only if the CRE was confirmed to be accessible within that cell type. This approach ensures that while our search space leverages the full statistical power of the atlas, the reported regulatory links for each cell type are strictly confined to the active chromatin landscape of that cell type.

## Results

### Database content overview

**Figure 2:**
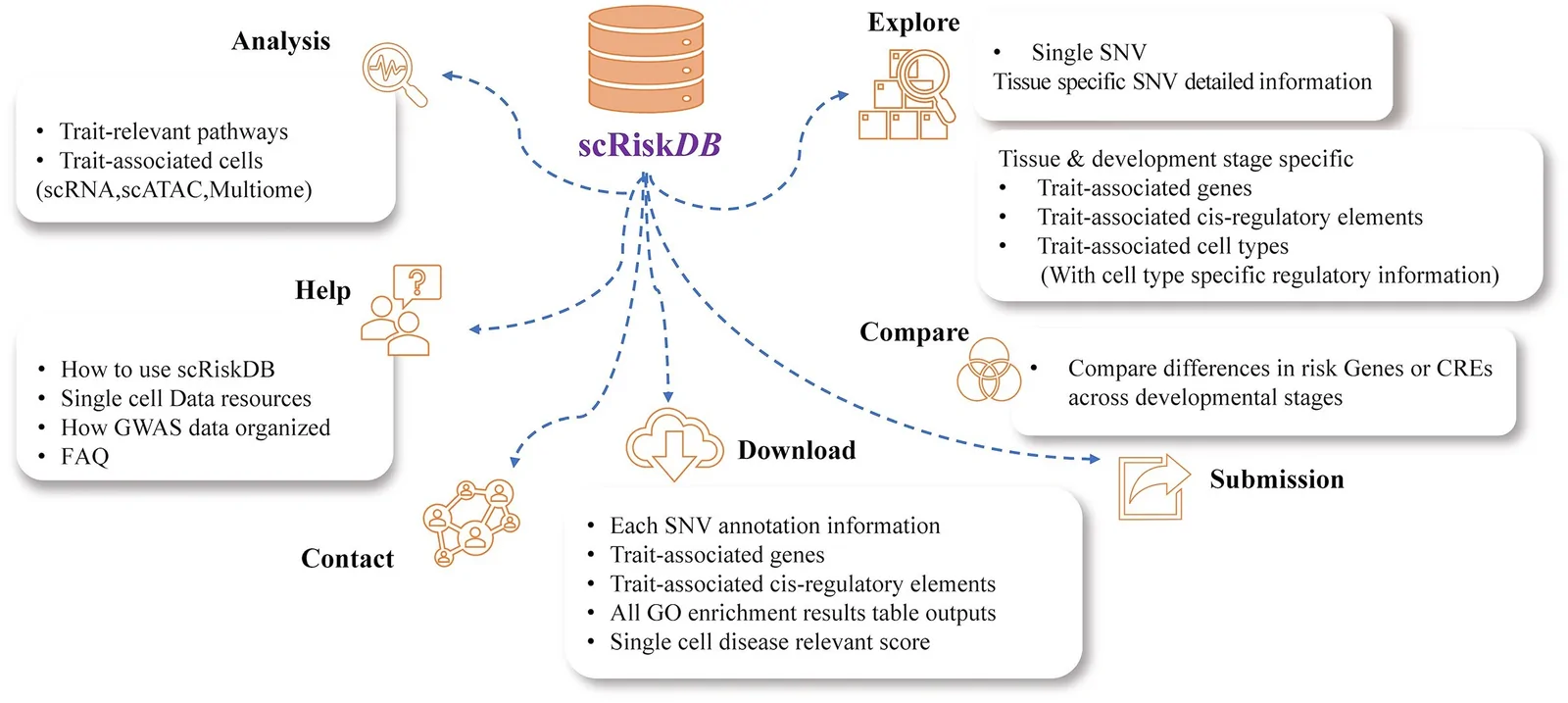
Database contents. The multiple functions supported by scRiskDB and the specific entries contained in each function.

Figure 2 outlines the overall content and key functionalities of scRiskDB. The platform allows users to search, explore, compare, analyse, and download trait-associated information across multiple biological levels, including variants, genes, CREs, cell types, and pathways. Users can perform cell-type-specific analyses using various single-cell omics datasets or conduct pathway analysis based on their research needs. Interactive tools are provided to compare risk genes and regulatory elements across different developmental stages. All result pages offer downloadable outputs to facilitate further analysis. A help section is also available to assist users in understanding the data structure and effectively navigating the platform.

### Database navigation

**Figure 3:**
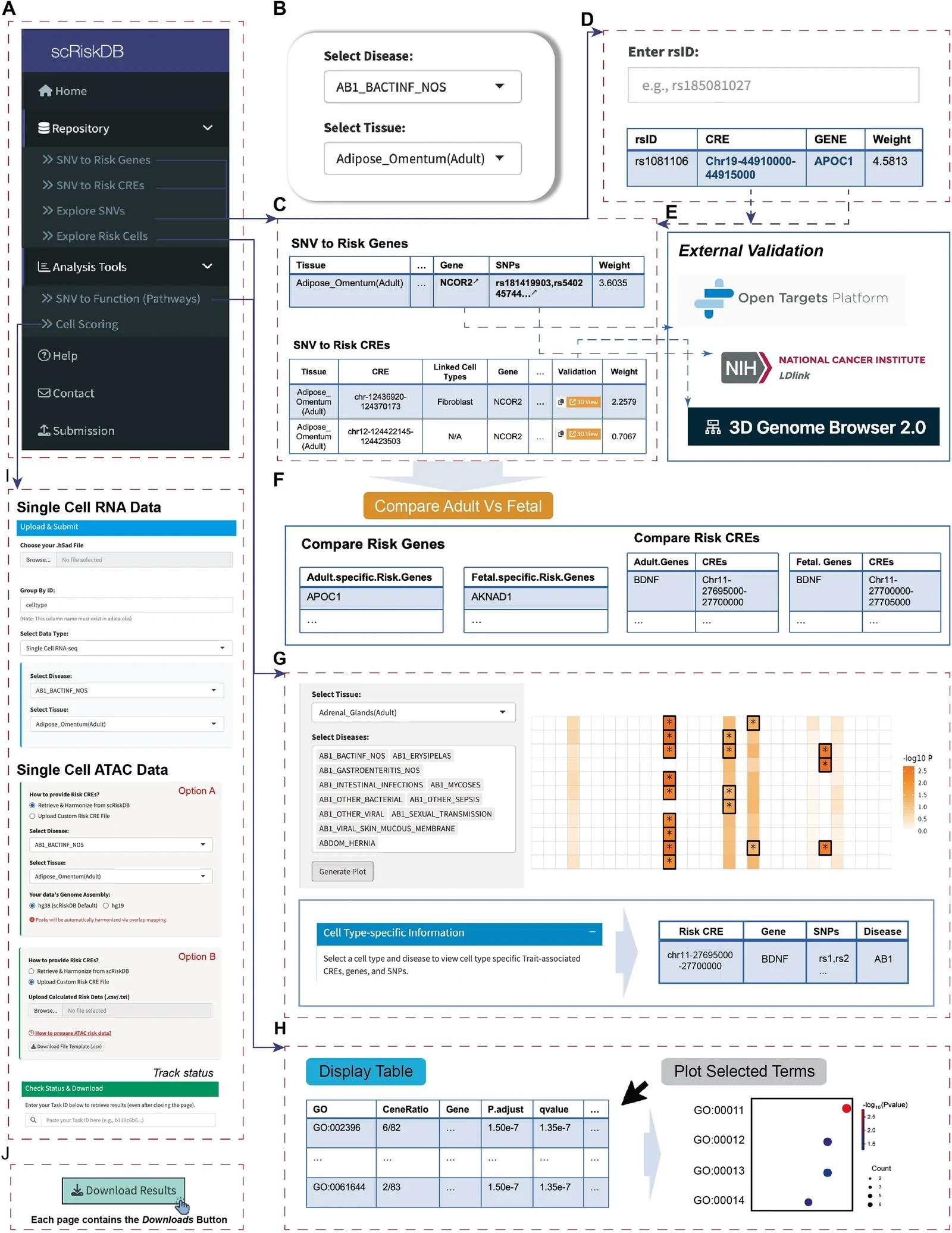
Main function navigation of scRiskDB. (A) Main navigation bar of scRiskDB. (B) Drop-down menus for selecting tissues and cell types in each exploration module. (C) Example output tables from the *SNV to Risk Genes* (top) and *SNV to Risk CREs* (bottom) section. (D) The *Explore SNVs* page allows users to input rsID to retrieve SNV annotations across tissues and diseases. (E) The external validation resources. (F) Comparison results across developmental stages, highlighting stage-specific genes (left) and gene–CRE pairs (right). (G and H) Two built-in analysis tools within *Analysis Tools* section: pathway enrichment analysis and cell-level scoring. (I) Example of selecting and querying status in *Cell Scoring* analysis section. (J) Download options are available for all subpages.

Figure 3 shows the detailed navigation features and user interface of scRiskDB, which has been designed to support flexible exploration and efficient analysis of multi-level trait-associated risk data. The platform provides a workflow that enables users to navigate from variant-level information up to cell-type-specific insights.

#### Explore from SNV to cell-level resolution

Users can start their query by selecting a disease and tissue of interest (Fig. 3B) on any explore subpage. Exploration can begin with tissue-specific genes or CREs by browsing through the relevant sub detailed pages (Fig. 3C). Alternatively, query can begin with a specific SNV, users can directly input a variant identifier (Fig. 3D) to retrieve associated regulatory cascades. SNVs listed in the interactive results table is hyperlinked to the dbSNP database [19] for external reference. To enable efficient multi-level exploration, the interface implements interactive cross-linking between variant-, CRE-, and gene-level annotations. Selecting a gene or CRE from the SNV results automatically redirects users to the corresponding detailed view, where the selected entry is highlighted.

Beyond internal annotations, scRiskDB integrates external functional resources to provide additional biological context (Fig. 3E). For risk genes, direct links to Open Targets [20] allow users to explore aggregated disease–gene association evidence from diverse genetic, functional, and literature-based sources. Integration with LDexpress [21] enables users to examine expression quantitative trait locus (eQTL) associations for variants in linkage disequilibrium with the queried SNVs. For candidate CREs, the exploration module includes a ‘Validation’ feature that provides direct access to the 3D Genome Browser [22], enabling visualization of chromatin interaction data, such as Hi-C loops, at the queried loci (±100 kb). In addition, CRE results include cell-type accessibility annotations, indicating the cellular contexts in which the regulatory elements are predicted to be active (Fig. 3C).

A particularly useful feature is to rank elements by risk weight score to prioritize key genes, or CREs. Additionally, users can compare trait-associated gene or CRE profiles across developmental stages directly within the detailed pages (Fig. 3F). Specifically, the gene page presents developmental stage-specific risk genes, while the CRE page displays specific risk-associated gene–CRE pairs, enabling users to investigate how disease risk may vary across developmental time points.

For cell-level exploration, users can select a tissue of interest along with one or more associated diseases to generate a heat map highlighting potential disease-relevant cell types (Fig. 3G). Statistically significant associations are marked with asterisks, helping users quickly identify the most relevant cell populations. Furthermore, a drop-down menu allows users to select specific cell types with a disease and view their associated CREs and risk genes, providing valuable insights into cell-type-specific regulatory mechanisms.

#### Easy online analysis functions

scRiskDB provides user-friendly online analysis tools, including a built-in module for pathway enrichment analysis. Users can perform Gene Ontology (GO) enrichment based on selected tissues and diseases within the Analysis Tools section (Fig. 3A). The results are first presented in a sortable interactive table, where users can select GO terms of interest, which are then visualized as bubble plots for intuitive interpretation (Fig. 3H). And the GO terms are hyperlinked to QuickGo [23] website for users to browse the GO and its associated annotations. In addition, scRiskDB supports user-uploaded single-cell RNA-seq or ATAC-seq data to calculate cell-level disease relevance scores (Fig. 3I). To utilize this function, users are required to upload a quality-controlled .h5ad file containing the count matrix, with the cell-type annotations (or other grouping variables) stored in the obs metadata. If single-cell RNA-seq data are selected, scRiskDB will automatically retrieve the corresponding risk gene list based on the chosen tissue and disease to perform the analysis. For single-cell ATAC-seq, scRiskDB provides two alignment options. Using Auto-Harmonization, users can simply select a supported tissue, disease, and genome assembly (hg19/hg38). The backend then automatically retrieves the Risk CREs, applies a liftover if necessary, and maps them to the uploaded peak set. Alternatively, for unsupported traits, users can perform a Custom Upload. To assist with this, we provide a downloadable CSV template and a direct link to Help page for generating reproducible risk files. For multiome datasets, users can select either the RNA or ATAC to proceed with the analysis. For multiome datasets, users can select either the RNA or ATAC to proceed with the analysis.

#### Supporting data download

Each result page includes a dedicated ‘Download Results’ button (Fig. 3J), which allows users to export their search results for offline use. The downloadable content includes SNV-gene/CRE mappings, pathway analysis tables, and cell-type-specific trait annotation results. This function facilitates integration with downstream analyses or publication-ready visualizations.

#### Comprehensive help page

The scRiskDB Help section (Fig. 3A, ‘Help’ panel) provides clear, step-by-step instructions in the side navigation panel for common tasks and a Frequently Asked Questions section for common user queries. For the online cell-level disease score analysis module, detailed tutorials, template files, and helper scripts have been integrated to enable the formatting of user-provided single-cell data and risk annotations. This section ensures that even first-time users can quickly understand and navigate the platform.

### Case study

**Figure 4:**
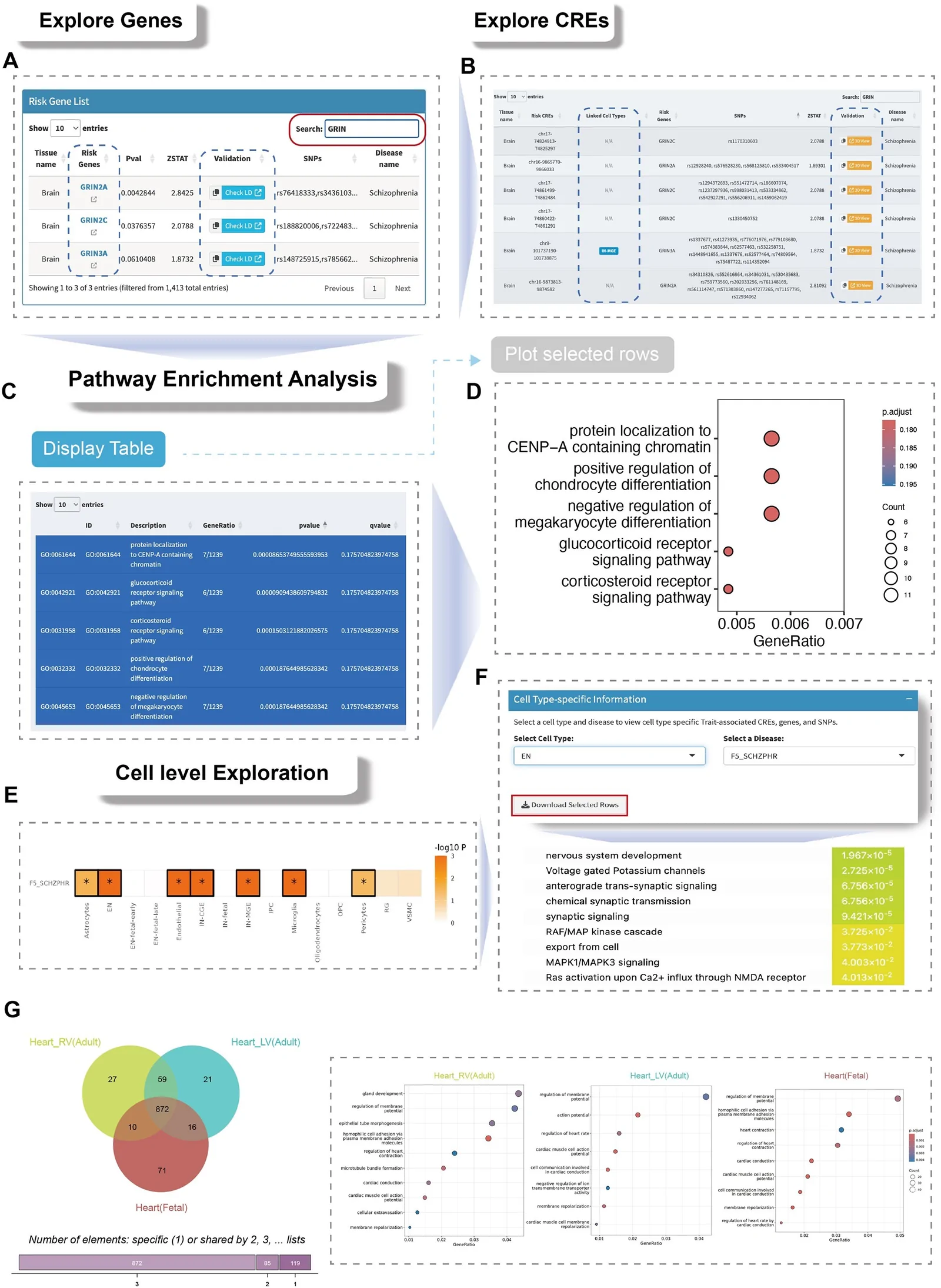
Case studies demonstrating multi-level regulatory exploration using scRiskDB. (A) Gene exploration in brain tissue identifies schizophrenia (SCZ)-associated GRIN family genes (e.g. GRIN2A) and provides integrated access to external validation resources such as Open Targets and LDexpress. (B) The *Explore CREs* module identifies specific *cis*-regulatory elements linked to the prioritized risk genes with cell-type-specific information and 3D genome resources. (C) Pathway Enrichment Analysis of SCZ risk genes highlights stress-related and epigenetic signalling pathways. (D) Interactive GO term visualization allows users to select specific biological processes and generate publication-ready plots. (E) Cell-level associations identify excitatory neurons (ENs), astrocytes, and oligodendrocyte precursor cells (OPCs) as SCZ-relevant cell types. (F) Risk CREs in ENs are retrieved for download (Up) and further downstream analysis (Down). (G) Cross-tissue comparison of Atrial Fibrillation (AF) risk genes. A Venn diagram (left) shows the overlap of prioritized risk genes across adult right ventricular (RV), left ventricular (LV), and foetal cardiac tissues. The corresponding GO enrichment analysis (right) highlights the conserved electrophysiological core alongside tissue-specific remodelling pathways.

To demonstrate the use of scRiskDB, we first present a case study focused on schizophrenia (SCZ). We selected brain tissue due to the central role of brain-specific regulatory networks in the aetiology of psychiatric disorders. In scRiskDB, users can initiate exploration from multiple entry points, including risk genes (Fig. 4A) or candidate CREs (Fig. 4B) through interactive result tables that integrate disease association statistics, linked variants, and external annotation resources. Here, we demonstrate a gene-centred exploration strategy by focusing on the GRIN gene family, which has been extensively implicated in SCZ. Using the gene search function, we queried the GRIN family and identified several SCZ-associated members, including GRIN2A, GRIN2C, and GRIN3A, all of which have been previously reported to contribute to psychiatric disease susceptibility [24–28].

To support biological interpretation, the gene detail page in scRiskDB provides two integrated external functions (Fig. 4A). Using GRIN2A as an example, the Open Targets link enables users to systematically examine gene disease association evidence across multiple data sources. As shown in Supplementary Fig. S1A, GRIN2A is supported by diverse evidence types, including GWAS associations, gene burden studies, ChEMBL, and literature evidence curated in Europe PMC.

To further refine candidate functional variants linked to prioritized genes, scRiskDB provides a *Check LD* function that enables downstream exploration of potential regulatory mechanisms. This function takes the top 10 SNVs linked to the selected gene entry and redirects users to the LDexpress [21] interface. Users can then paste SNVs and specify required parameters by LDexpress (Supplementary Fig. S1B). The result shows that rs4782113 is in strong linkage disequilibrium with rs16966757 (*R*² *=* 0.86, *D' =* 0.98). Notably, the proxy variant rs16966757 exhibits significant eQTL associations with GRIN2A across multiple brain regions, including Brain–Putamen (basal ganglia), Brain–Spinal cord (cervical c-1), and Brain–Caudate (basal ganglia), with *P* values ranging from 6.4 × 10^−7^ to 2.7 × 10^−8^. Complete LDexpress results are provided in Supplementary Table S3.

Building on these variants and genes observations, users can further investigate the regulatory landscape by querying linked CREs using the Explore CREs module (Fig. 4B). To validate whether these predicted regulatory relationships are supported by higher-order chromatin organization, scRiskDB integrates a 3D genome visualization module. Visual inspection of human Hi-C data revealed a significant long-range chromatin interaction connecting a CRE (chr16:9 873 813–9 874 582) to the GRIN2A promoter region. Both loci are located within the same Topologically Associating Domain (TAD) (Supplementary Fig. S1C, yellow bar), satisfying known structural constraints for *cis*-regulatory interactions. This chromatin looping evidence provides additional support for the regulatory potential of the prioritized variant containing CRE. Finally, scRiskDB further enhances regulatory interpretation by incorporating cell-type resolved chromatin accessibility annotations. By integrated cell-type annotation links, users can identify candidate CREs exhibiting cell-type-specific accessibility patterns, enabling functional interpretation of regulatory variants within disease-relevant cellular contexts.

And it is an excellent try to use the Pathway Enrichment Analysis module to investigate the broader biological context of disease risk genes. Enriched pathways included glucocorticoid receptor signalling, corticosteroid signalling, and protein localization to CENP-A chromatin (Fig. 4C), suggesting roles in stress response and epigenetic regulation [29–32]. Other terms, like chondrocyte differentiation and megakaryocyte differentiation, may reflect developmental or immune-related aspects of SCZ [33]. To facilitate interpretation, users can interactively visualize selected GO terms and generate publication-ready plots to support downstream hypothesis generation or functional validation (Fig. 4D).

Lastly, we assessed the cell-type specificity of SCZ using the Cell Level Exploration module. Significant associations were observed in excitatory neurons (EN), astrocytes, and oligodendrocyte precursor cells (OPCs), corroborating previous findings of neuronal and glial involvement in SCZ [34] (Fig. 4E). By selecting EN as the target cell type, we retrieved a list of risk CREs, which can be downloaded for further analysis (Fig. 4F). To show the potential downstream applications of these results, we performed GO enrichment analysis on the genes linked to these CREs whether the genes themselves were previously identified as risk genes. The enriched terms revealed additional biological processes not directly highlighted by earlier analyses, suggesting that scRiskDB can also generate novel insights by uncovering regulatory networks and functional associations beyond established results [35–37].

Expanding beyond the dissection of cell-type-specific networks within a single organ system, scRiskDB enables systematic cross-tissue comparisons to resolve the spatial and temporal heterogeneity of complex diseases. We applied this approach to compare the regulatory landscapes of Atrial Fibrillation (AF) across right ventricular (RV), left ventricular (LV), and foetal cardiac tissues. As shown by the Venn diagram of AF risk genes (Fig. 4G), this comparison successfully identified a conserved electrophysiological core alongside tissue-specific remodelling modules underlying AF genetic susceptibility. Across all three tissues (Fig. 4G), prioritized AF risk genes consistently enriched for pathways related to cardiac muscle cell action potential, membrane repolarization, and regulation of membrane potential. This overlap indicates that the disruption of cardiac electrical excitability is a shared mechanism driving AF susceptibility, aligning with GWAS that link ion channel dysfunction to arrhythmogenesis [38, 39].

Beyond this core, tissue-specific regulatory programmes were evident. In the RV, AF risk genes enriched for the negative regulation of ion transmembrane transporter activity and cell communication involved in cardiac conduction, driven by RV-specific elements linked to genes such as *CDH1* and *DRD2*. This points to an RV-specific genetic contribution to fine-tuning impulse propagation and pacing stability.

In contrast, LV-associated AF risk genes showed enrichment for homophilic cell adhesion, microtubule bundle formation, and epithelial tube morphogenesis. Notably, the LV-specific gene set uniquely includes *PLN* and *TCAP*, which are critical for excitation–contraction coupling and sarcomere stability. This finding strongly supports the concept that ventricular structural remodelling and subclinical cardiomyopathies share a genetic basis with AF and can serve as an arrhythmogenic substrate [40, 41].

Finally, foetal cardiac tissue displayed distinct enrichment for the coordinated regulation of heart contraction and cardiac conduction. This is underscored by the exclusive prioritization of key early cardiac maturation and calcium-handling genes in the foetal module, including *CASQ2* and *KCNJ2*. This observation aligns with the developmental origins of arrhythmia, suggesting that early genomic programming of electrophysiological synchronization and excitation-contraction coupling establishes long-term susceptibility to AF [42, 43].

Overall, these case studies highlight how scRiskDB bridges the gap between a genetic variant and its regulatory, tissue, and cell-type-specific context, facilitating a deeper mechanistic understanding of complex traits.

## Conclusion

scRiskDB is a comprehensive and user-friendly database designed to systematically explore the regulatory impact of disease-associated variants across tissues, developmental stages, and single-cell resolution.

Compared to previous resources, scRiskDB offers a richer integration of variant-centred regulatory annotations and enables developmental and cell-type-specific risk element discovery through interactive visualizations and modular interfaces. The platform supports multiple exploration paths, from trait and tissue selection to variant-level queries and cell-specific analysis facilitating both hypothesis generation and in-depth mechanistic investigation.

While scRiskDB represents a significant step forward in linking genetic risk with functional regulatory landscapes, certain limitations remain. One key constraint lies in the incomplete availability of human tissues and developmental stages, which may limit the comprehensiveness of trait–tissue and trait–cell associations. Moreover, the disease-associated SNVs identified the from multi-marker method may not be causal, posing challenges for precise mechanistic interpretation. In future updates, we plan to incorporate newly developed algorithms to better prioritize causal variants, as well as to expand the coverage of tissues and cell types included in the database. Second, our *cis*-regulatory interactions were inferred at the tissue level to overcome the sparsity of single-cell data. While we filtered these interactions based on cell-type-specific chromatin accessibility, this represents a necessary condition for regulation rather than direct evidence of physical looping in each cell type. Therefore, the identified risk mechanisms should be interpreted as high-confidence putative candidates that prioritize functional potential within specific cellular contexts. We will also continuously expand the coverage of human tissues and developmental stages as new single-cell atlases are published. Importantly, we have established a multi-omics analytical framework within the current version. This scalable design ensures that scRiskDB is functionally equipped to rapidly ingest and harmonize emerging single-cell multiome datasets across other tissues in the future. Ultimately, we aim to actively incorporate user feedback to ensure the platform evolves dynamically alongside advancements in the field of complex trait genetics.

## Supplementary Material

baag048_Supplemental_Files

## Data Availability

scRiskDB is accessible via http://scriskdb.cn/ and the shiny-based framework code for web builds is available on GitHub https://github.com/gefeiZ/scRiskDB. To facilitate reproducibility and local deployment, all associated data have been deposited in Zenodo under two separate records: the processed single-cell matrices and supplementary material are available at https://zenodo.org/records/18765434, while the lightweight example MySQL database for local server configuration is available at https://zenodo.org/records/21310670.
